# Lysinuric Protein Intolerance and Its Nutritional and Multisystemic Challenges in Pregnancy: A Case Report and Literature Review

**DOI:** 10.3390/jcm12196405

**Published:** 2023-10-08

**Authors:** Adriana Pané, Camila Milad, Marta Santana-Domínguez, Núria Baños, Cristina Borras-Novell, Gerard Espinosa, Laura Magnano, Meritxell Nomdedeu, Pedro Juan Moreno-Lozano, Frederic Cofan, Mercè Placeres, Rosa Maria Fernández, Judit García-Villoria, Glòria Garrabou, Irene Vinagre, Laura M. Tanner, Cristina Montserrat-Carbonell, Maria de Talló Forga-Visa

**Affiliations:** 1 Endocrinology and Nutrition Department, Hospital Clínic, Villarroel 170, 08036 Barcelona, Spain; 2 Adult Inborn Errors of Metabolism Unit, Hospital Clínic, Villarroel 170, 08036 Barcelona, Spain; 3 Centro de Investigación Biomédica en Red de la Fisiopatología de la Obesidad y Nutrición (CIBEROBN), Instituto de Salud Carlos III (ISCIII), 28029 Madrid, Spain; 4 Neonatology Department, BCNatal (Barcelona Center for Fetal and Neonatal Medicine), Hospital Clínic, Villarroel 170, 08036 Barcelona, Spain; 5 Fundació Clínic per la Recerca Biomèdica (FCR), Institut d’Investigacions Biomèdiques August Pi Sunyer (IDIBAPS), 08036 Barcelona, Spain; 6 Autoimmune Diseases Unit, Hospital Clínic, Villarroel 170, 08036 Barcelona, Spain; 7 Department of Hematology, Hospital Clínic, Villarroel 170, 08036 Barcelona, Spain; 8 Internal Medicine Department, Hospital Clínic, Villarroel 170, 08036 Barcelona, Spain; 9 Asociación Española para el Estudio de los Errores Congénitos del Metabolismo (AECOM), 28221 Majadahonda, Spain; 10 Renal Transplantation and Nephrology Department, Hospital Clínic, Villarroel 170, 08036 Barcelona, Spain; 11 Pharmacy Department, Hospital Clínic, Villarroel 170, 08036 Barcelona, Spain; 12 Biochemistry and Molecular Genetics Department, Biomedical Diagnostic Center, Hospital Clínic, Villarroel 170, 08036 Barcelona, Spain; 13 Centro de Investigación Biomédica en Red de Enfermedades Raras (CIBERER), Instituto de Salud Carlos III (ISCIII), 28029 Madrid, Spain; 14 Inherited Metabolic Diseases and Muscle Disorders Laboratory, FCRB-IDIBAPS and Faculty of Medicine and Heath Sciences, University of Barcelona, 08036 Barcelona, Spain; 15 Fetomaternal Medical Center and Department of Clinical Genetics, Helsinki University Hospital, Department of Medical and Clinical Genetics, University of Helsinki, 00251 Helsinki, Finland

**Keywords:** lysinuric protein intolerance, nutrition, hyperammonemia, pregnancy, inborn error of metabolism, rare disease

## Abstract

Lysinuric protein intolerance (LPI) is a rare inborn error of metabolism (IEM), classified as an inherited aminoaciduria, caused by mutations in the SLC7A7 gene, leading to a defective cationic amino acid transport. The metabolic adaptations to the demands of pregnancy and delivery cause significant physiological stress, so those patients affected by IEM are at greater risk of decompensation. A 28-year-old woman with LPI had experienced 3 early miscarriages. While pregnancy was finally achieved, diverse nutritional and medical challenges emerged (food aversion, intrauterine growth restriction, bleeding risk, and preeclampsia suspicion), which put both the mother and the fetus at risk. Moreover, the patient requested a natural childbirth (epidural-free, delayed cord clamping). Although the existence of multiple safety concerns rejected this approach at first, the application of novel strategies made a successful delivery possible. This case reinforces that the woman’s wish for a non-medicated, low-intervention natural birth should not be automatically discouraged because of an underlying complex metabolic condition. Achieving a successful pregnancy is conceivable thanks to the cooperation of interdisciplinary teams, but it is still important to consider the risks beforehand in order to be prepared for possible additional complications.

## 1. Introduction

Lysinuric protein intolerance (LPI) is a rare disorder classified in the Inborn Error of Metabolism (IEM) group, with an autosomal recessive inheritance. It is caused by mutations in the SLC7A7 gene, which encodes the y+LAT–1 protein [y(+) L–type amino acid transporter 1]. A defective y+LAT–1 protein results in an abnormal cationic amino acid (AA) transport, leading to deficient gastrointestinal absorption and urine loss of arginine, ornithine, and lysine [1,2]. The reduced availability of arginine and ornithine compromises the normal functioning of the urea cycle (the major pathway for the disposal of nitrogen in humans) as both AA act as urea cycle intermediates [3].

Although LPI is characterized by protein intolerance and failure to thrive, it represents a severe multisystemic disorder in which almost any organ can be affected: gastrointestinal tract (vomiting and chronic diarrhea), lungs (pulmonary alveolar proteinosis (PAP)) and kidneys (tubulopathy, proteinuria, and renal failure). Hematological defects (anemia, leukopenia, thrombocytopenia, hemophagocytic lymphohistiocytosis/macrophagic activation syndrome (HLH/MAS)), altered immune response (autoimmune disorders, deficient B–cell function), and hypercholesterolemia/hypertriglyceridemia are also usual findings [1].

Therapeutical management is based on a low-protein diet, supplemented with citrulline (lysine can also be considered), L-carnitine, vitamins, and other micronutrients if necessary, along with nitrogen-scavenging drugs [1,2,4].

LPI integrative treatment has helped females to reach their reproductive age. As pregnancy and delivery constitute significant stressors, any woman is at greater risk of metabolic decompensation at this time [5,6]. When an IEM coexists, this risk becomes even greater [7]. A limited number of case reports on LPI in pregnancy and only one cohort study from Finland have been published thus far [5,8,9,10]. However, none of the reported cases have simultaneously faced: (1) the classical, but challenging, both medical and nutritional disorders of LPI in the pregnancy setting and (2) the patient’s desire for natural childbirth despite suffering a complex metabolic disorder. To contribute to addressing this gap in the literature, we herein present the introduction of novel strategies to improve dietary control and intrapartum ammonia levels monitoring in a pregnant woman with LPI who developed threatening complications but expressed her desire for a natural delivery.

## 2. Case Report

We present a 28-year-old woman who was diagnosed with genetically confirmed LPI in her country of origin when she was 4 years of age. Before knowing LPI diagnosis, she had required a total of three admissions to a Pediatric Hospital due to hyperammonemia. Her mother did not report any developmental concerns during childhood. In fact, she was able to attend university and successfully complete a degree.

### 2.1. Preconception Phase

She was first admitted to our center at the age of 19. No major clinical consequences were known except for osteoporosis. She was recommended treatment with sodium benzoate and citrulline according to current evidence [1]. However, she had a completely irregular clinical follow-up. Of note, although her ammonia levels tended to be under target, the concentration of plasmatic glutamine was above 900 µmol/L.

The patient had been trying to conceive for 2 years and had experienced three early miscarriages. She also presented non-specific blood markers of autoimmunity, including antinuclear antibodies, anti-Ro/SSA antibodies, and hypocomplementemia without fulfilling the classification criteria of any systemic autoimmune diseases. Repeated determinations of antiphospholipid antibodies (aPL) were negative. Despite aPL negativity and given the previous adverse pregnancy outcomes and autoimmunity markers, a tentative diagnosis of seronegative obstetric antiphospholipid syndrome was made. She was therefore advised to start treatment with preconceptional low-dose aspirin and prophylactic low molecular weight heparin (LMWH) once pregnancy was confirmed. Prednisone was also added and de-escalated later. Due to anti-Ro/SSA antibody positivity, 200 mg/day of hydroxychloroquine was started to prevent fetal congenital heart block.

### 2.2. Pregnancy

**Figure 1:**
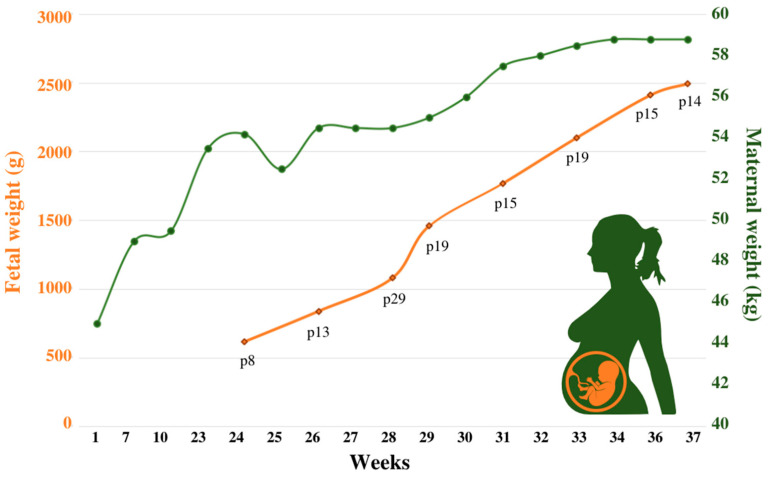
Maternal and fetal weight throughout pregnancy.

The main challenge during the first trimester was the patient’s poor dietary intake due to recurrent vomiting (coinciding with acute bronchitis) and aversion to high-protein foods. To solve this situation, which had resulted in the deficiency of multiple essential amino acids (EAA) (Table 1) and insufficient weight gain (Figure 1), we directed her to prioritize natural protein intake and designed a *shake* (the composition is presented in Table 2) to be to distributed between three intakes. With regards to bronchitis, oral azithromycin plus bronchodilator therapy were sufficient to resolve the episodes.

In the second/third trimesters, the composition of the *shake* was modified: (a) a carbohydrate powder supplement replaced the dual formula (energy source of carbohydrate and fat) used in the first trimester to correct for the worsening lipid profile and, (b) a powdered EAA supplement was introduced aiming to improve the low weight percentile of the fetus and to overcome the mother’s EAA deficiencies (Table 2). Although the maternal weight before pregnancy was 45.0 Kg, under this tailored-nutritional strategy she reached 49.5 Kg at the beginning of the second trimester. The mother’s body weight progressively increased until reaching a maximum of 58.8 Kg at the end of pregnancy (Figure 1).

Supplementation with L-citrulline 3–9 g/day, and treatment with sodium benzoate 4–6 g/day, as a nitrogen-scavenging drug, were maintained and adjusted according to ammonia blood levels and urinary orotic acid (Table 1).

The recommended daily intake of vitamins and minerals for pregnant women [11] was ensured through specifically designed commercial supplements. Details concerning the nutritional adjustments are presented in Table 2.

Serum creatinine (Cr) was regularly assessed during the first and second trimesters. Although the estimated glomerular filtration rate (eGFR) calculated with the CKD–EPI formula from Cr was always ≥90 mL/min/1.73 m^2^, concerns regarding its applicability emerged due to her poor nutritional status. Therefore, urinary protein measurements (both in 24 h urine collection and spot urine samples), Cr clearance, and eGFR from cystatin C/Cr-derived formulas were also checked in the third trimester. No clinically significant alterations were documented, except for mild proteinuria (Table 3).

Anemia and thrombocytopenia occurred in the second trimester and, gradually deteriorated. Although both concerns are common in patients with LPI, to exclude other reasonable and treatable causes, the concentration of dietary factors involved in erythropoiesis was measured. Ferritin remained high, but relatively stable throughout pregnancy. Folic acid and vitamin B12 levels were normal, but hemoglobin reached a nadir value of 72 g/L. Therefore, the patient received a total of three red blood cell (RBC) transfusions. The platelet count checked with EDTA samples progressively decreased (nadir value of 67 × 10^9^/L). However, when the platelet count was tested with citrate, multiple platelet aggregates were observed. To better assess these blood cell abnormalities, a blood smear was performed revealing RBC anisopoikilocytosis, polychromasia, basophilic stippling, and the presence of large platelets. In addition, heparin-induced thrombocytopenia was ruled out (anti-platelet factor 4 antibody levels) and the concentration/activity of those factors directly involved in the coagulation pathways was measured. As no alterations were detected and, in accordance with the patient’s will, both aspirin and LMWH were maintained under close and weekly supervision by the hematologist’s team. Further details regarding the hematologic, hemolytic, and coagulation evaluation are presented in Table 4.

The sFlt–1/PlGF ratio was checked from 35.0 weeks onwards to track PE risk, with the highest recorded value being 69.7 (week 35.0). PE will be diagnosed when the sFlt-1/PlGF ratio is >85 (<34 weeks) or >110 (between weeks 34 and 36.6). PE will be excluded when the sFlt–1/PlGF ratio is <38. When the sFlt-1/PlGF ratio is between 38–85 or 38–110 depending on gestational age, it should be monitored weekly. At that time, no other signs or symptoms of PE were present apart from thrombocytopenia and elevated liver enzymes, which could be explained by her underlying pathology. Subsequent monitoring of angiogenic factors showed a progressive decrease, until finally normalizing.

### 2.3. Delivery and Post-Partum

The patient was admitted to the maternity hospital at 36.6 weeks due to high blood pressure levels and mild proteinuria. The sFlt–1/PlGF ratio at admission was 9.43, a value that rules out PE in the general pregnant population. However, since hypertension and proteinuria were present and due to concerns regarding the prognostic value and interpretation of the sFlt–1/PlGF ratio in the presence of her metabolic disease, the patient was advised to undergo labor induction, which was started with vaginal dinoprostone. To promote the progress of labor, an oxytocin infusion was added and a single dose of 900 mg intravenous tranexamic acid for hemorrhage prevention was administered.

A glucose infusion (glucose 10% at 62 mL/h), along with oral citrulline and sodium benzoate, was administered throughout the active labor (6 h) and until the patient was able to tolerate oral feeding. The pain was managed with nitrous oxide and remifentanil since she did not desire spinal anesthesia.

Blood ammonia concentration was checked at the time of admission and every 8 h once active labor started. Since plasma ammonia measurement was not directly available in the maternity hospital and immediate refrigerated transport to the general adult’s hospital was required, bedside capillary blood ammonia (expressed in µg/dL) was assayed as a proxy to plasmatic levels (µmol/L) and quantified with a portable handheld meter (PocketChem BA PA–4140, Arkray Inc., Kyoto, Japan). Ammonia levels (both plasmatic and capillary) were consistently lower than 80 µmol/L, being the upper reference range for healthy adults of 50 µmol/L.

**Figure 2:**
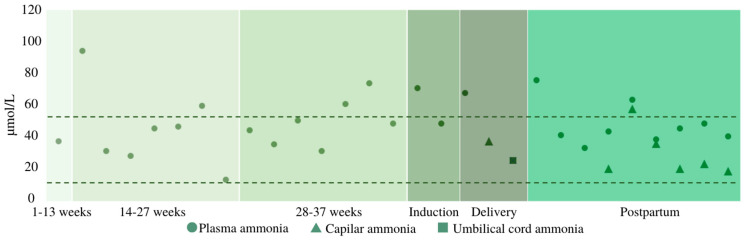
Ammonia levels monitoring. The area within the dotted line indicates [ammonia] normality for healthy adults (9.0–50.0 µmol/L).

Although efforts were made to allow a safe delayed cord clamping, since ammonia levels were not within the normal range (while adequate considering the urea cycle disorder (UCD) setting), the patient accepted a “semi-delayed” (before 1 min) cord clamping to avoid a potential ammonia transfer to the infant. At the time of birth, the mother’s capillary ammonia was 65 µg/dL (38 µmol/L) and the neonatal cord ammonia was 26 µmol/L (Figure 2).

After 24 h of labor induction, she delivered a healthy male newborn with an Apgar score of 9/10/10 and an adequate gestational age birthweight of 2495 g, corresponding to the 14th percentile. The newborn’s capillary ammonia could not be evaluated because of technical issues until 48 h after the delivery, once breastfeeding had been initiated, it was 38 µg/dL (22 µmol/L).

No obstetric intrapartum or immediate postpartum complications occurred. The newborn showed non-isoimmune hyperbilirubinemia that was appropriately resolved with phototherapy. The newborn’s blood spot screening was strictly normal. Both plasmatic and urinary aminograms were performed without detecting any alterations suggestive of LPI.

The infant was exclusively breastfed for 6 months. Because of the known augmented women’s caloric demands during breastfeeding, the patient was recommended to increase her daily caloric intake until reaching a minimum of 2000 kcal/day [7]. Additionally, she was kept on the same dietary supplementation and pharmacological treatment that had been established in the third trimester.

## 3. Discussion

IEM were once considered exclusively pediatric conditions; however, they currently represent a growing challenge in adult medicine. Indeed, more patients with IEM are now reaching the childbearing age and pursuing pregnancy [11,12]. Furthermore, as independent adults, patients are the ones who actively take control of their diet (not their parents anymore) and so, tailored and supervised dietary management becomes essential for good health and well-being.

The clinical characteristics of patients with LPI go beyond the classical UCD [13], suggesting an increased risk for maternal complications during pregnancy and delivery [1,2]. There are a few reported cases of LPI and pregnancy [5,8,9,10] that shared some of our main medical challenges but did not face the patient’s wish to have a natural delivery.

In our patient, metabolic needs could be met through a tailored dietetic plan including a *shake* specially designed to avoid both essential and non-essential AA deficiencies. As reported in the Finnish cohort, plasma leucine and isoleucine were constantly decreased, along with cationic AA [5]. Meanwhile, in contrast to healthy pregnancies [14], methionine decreased across gestation while no significant changes were observed in phenylalanine levels. It should be stressed that although ammonia concentration was within an acceptable range, plasma glutamine was further above the upper limit of normal. Indeed, in certain patients with UCD, glutamine may be chronically elevated without high ammonia levels at the same time point indicating metabolic instability. Even if glutamine does not cause brain edema as in acute hyperammonemia, glutamine itself is neurotoxic [15,16]. Among nitrogen scavengers, sodium phenylbutyrate and glycerol phenylbutyrate bind directly to glutamine allowing better clearance; on the other hand, sodium benzoate binds to glycine [17]. However, since the use of benzoate in pregnancy is more widespread, in accordance with the patient, her usual pharmacological treatment was not modified.

Bronchitis episodes compromised the patient’s calorie intake. Even though PAP could not be ruled out, a deterioration of the phagocytic function leading to an abnormal immune response might have contributed to her respiratory symptoms [2].

Renal involvement is another main concern in patients with LPI, but in our case, renal function appeared to be preserved. Glomerular filtration rate (GFR) was also estimated using cystatin C to avoid the limitations derived from Cr, observing a GFR appropriate to pregnancy-related hyperfiltration [18]. Non-clinically significant mild proteinuria emerged in the third trimester but promptly resolved afterward.

With regards to liver enzymes, transaminases tended to increase after the second trimester and remained slightly elevated 1 year after pregnancy. A similar pattern was observed for both cholesterol and triglycerides.

As already presented in other cases, anemia and thrombocytopenia emerged [5,8,10]. Although thrombocytopenia and anemia are characteristic of LPI [19], specific analyses were performed to exclude additional treatable causes. The main nutritional factors associated with erythropoiesis were in the reference range. Notably, serum zinc concentration markedly exceeded the upper limit of normality in the first trimester. It has been hypothesized that defects in the metabolism of calprotectin, the major calcium- and zinc-binding protein of phagocytes, may be related to this condition [20]. Considering another plausible cause of anemia, it is known that glycine takes an active part in hemoglobin synthesis [21] and that benzoate decreases its levels [22]. We therefore systematically assessed glycine levels, but no defects could be observed. Attending thrombocytopenia management, in light of the PE risk and the previous tentative diagnosis of seronegative obstetric antiphospholipid syndrome, the need to maintain both aspirin and LMWH was reviewed.

Moving onto the PE risk, an imbalance between proangiogenic (i.e., PlGF: Placental Growth Factor) and antiangiogenic factors (i.e., sFlt–1: Soluble fms-like tyrosine kinase-1), namely an increased sFlt–1/PlGF ratio, results in a net antiangiogenic state which favors the development of placental dysfunction [23]. The sFlt–1/PlGF ratio within the reference range (<38) has shown a very high negative predictive value for the short-term prediction of PE and is used to rule out an imminent threat in a pregnant woman with clinical or analytical suspicion of PE [24]. However, an elevated ratio does not necessarily indicate an increased risk as it can be caused by other factors (e.g., the presence of a small-for-gestational-age fetus). Angiogenic factors within the normal range at the time of the onset of arterial hypertension make the diagnosis of PE very unlikely in this patient. Moreover, other signs of PE, such as thrombocytopenia, mild proteinuria, and elevated liver enzymes could be explained by her underlying pathology and appeared earlier in gestation.

The monitorization of ammonia levels during delivery, as well as its concentration in the umbilical cord and the newborn, has not previously been reported in LPI. In our case, as plasmatic ammonia determination was not readily available in the maternity hospital, capillary blood ammonia was also measured with a portable meter. This analyzer uses a single wavelength reflectance method and special reagent strips, with a measurement range from 10 to 400 µg/dL (conversion factor from µmol/L to µg/dL = 0.587). While a good correlation between plasmatic and capillary ammonia could not be established, since many external factors could have obscured the relationship [25], capillary levels constituted a good aid in tracking ammonia oscillations [26]. It has been described that ammonia is produced by deamination within both the fetus and the placenta. However, the arterial umbilical ammonia concentration exceeds the venous umbilical concentration, indicating a net ammonia production by fetal tissues in humans [27]. A better knowledge of ammonia levels evolution intrapartum could help to determine the plausibility of delayed cord clamping in IEM.

The main strength of this case report is the accurate description of the nutritional and medical interventions adopted to respect low-intervention natural childbirth in the presence of multiple challenges that may interfere with the course of any pregnancy. Of note, new hypotheses regarding ammonia and glutamine oscillations throughout pregnancy and delivery can be developed, along with the possibility of testing bedside capillary blood ammonia. However, the inherent limitations of any case report should also be acknowledged; such as the lack of ability to generalize and the danger of over-interpretation.

To conclude, careful nutritional and pharmacological treatment under the surveillance of integrative teams is crucial to ensure both maternal well-being and fetal development in any woman, especially when potentially threatening medical conditions such as LPI coexist [7]. This case reinforces that the woman’s wish for natural childbirth should not be automatically discouraged because of an underlying complex metabolic disorder. Although achieving a successful pregnancy is conceivable thanks to the cooperation of interdisciplinary teams, it is still important to consider the risks beforehand in order to be prepared for possible additional complications.

## Figures and Tables

**Table 1:** Laboratory tests before, during, and after pregnancy.

	Reference Values	Before Pregnancy	1st Trimester [1–13 Weeks]	2nd Trimester [14–27 Weeks]	3rd Trimester [28–37 Weeks]	1–Year after Pregnancy
**Urea cycle evaluation**						
Ammonia (µmol/L)	**9–50**	35	38	28	54↑	24
Ur orotic acid (mmol/mol)	**0.4–5.1**	---	---	3.9	2.7	1.4
**Plasma amino acids**						
Arg (µmol/L)	**15–140**	22.5	25.4	12.3↓	12.8↓	19.9
Cit (µmol/L)	**18–60**	25.3	30.3	18.0	25.6	73.5
Iso (µmol/L)	**40–100**	55.4	48.6	23.7↓	24.0↓	43.2
Leu (µmol/L)	**80–175**	84.2	100.1	72.8↓	41.5↓	91.6
Lys (µmol/L)	**100–280**	32.3↓	53.0↓	18.0↓	14.3↓	53.7↓
Met (µmol/L)	**20–40**	32.4	27.1	14.9↓	17.8↓	25.9
Gln (µmol/L)	**300–850**	1778.0↑	1088.9↑	904.4↑	593.7	1052.6↑
Glu (µmol/L)	**10–190**	39.1	27.3	48.7	27.1	97.7
Gly (µmol/L)	**130–350**	520↑	421↑	280	229	424↑
Orn (µmol/L)	**40–140**	15.8↓	21.1↓	10.1↓	12.0↓	15.5↓
Phe (µmol/L)	**30–100**	32.4	37.7	30.0	23.1↓	35.6
Thr (µmol/L)	**60–200**	117.5	132.4	96.5	57.2	107.9
Tyr (µmol/L)	**40–100**	34.4↓	40.0	25.3↓	11.0↓	43.4
Trp (µmol/L)	**30–120**	23.3↓	21.0↓	16.6↓	10.7↓	22.8↓
Val (µmol/L)	**140–340**	226.3	174.1	118.1↓	94.9↓	180.7
**Nutritional status**						
Albumin (g/L)	**34–48**	37	40	31↓	27↓	43
Cholesterol (mg/dL)	**<200**	117	244↑	154	238↑	200
Triglycerides (mg/dL)	**<150**	131	199	161	217↑	87
Free carnitine (µmol/L)	**18.1–57.0**	31.5	21.9	25.5	14.9↓	30.6
Magnesium (mg/dL)	**1.8–2.6**	1.3↓	1.8	1.4↓	1.3↓	1.4↓
Calcium (mg/dL)	**8.5–10.5**	8.6	9.2	8.3	8.2	8.8
**General renal function tests**						
Cr (mg/dL)	**0.3–1.3**	0.45	0.58	0.35	0.44	0.44
eGFR (CKD–EPI [Cr]) mL/min/1.73 m^2^	**≥90**	136	127	144	136	130
**Liver function tests**						
AST (U/L)	**5–40**	48↑	67↑	53↑	107↑	121↑
ALT (U/L)	**5–40**	20	35	22	53↑	63↑
ɣ–GT (U/L)	**5–40**	7	18	14	34	16

↓ Below and ↑ above the reference range. The median value for each trimester is represented: blood tests were performed every 4 months, except for the 2nd and 3rd trimesters (every 2 months). The 3rd trimester has an end at week 37.0 because labor was induced. ɣ–GT: ɣ–glutamyl transferase; ALT: alanine aminotransferase; Arg: arginine; AST: aspartate aminotransferase; Cit: citrulline; Cr: creatinine; eGFR: estimated glomerular filtration rate; Gln: glutamine; Glu; glutamic acid; Gly: glycine; Iso: isoleucine; Leu: leucine; Lys: lysine; Met: methionine; Orn: ornithine; Phe: phenylalanine; Thr: threonine; Trp: tryptophan; Tyr: tyrosine; Val: valine; Ur: urinary.

**Table 2:** Nutritional dietary plan before and throughout pregnancy.

			Before Pregnancy	1st Trimester [1–13 Weeks]	2nd Trimester [14–27 Weeks]	3rd Trimester [28–37 Weeks]
**Nutritional products**	**L-Citrulline** (g/day) (g/intake)		*Erratic consumption*	3 (1–1–1)	6 (2–2–2)	9 (3–3–3)
	***Shake***	**Multifruit juice** (mL/day)	---	1000	1000	1000
		**MCT oil** (mL/day) (mL/intake)	---	15 (5–5–5)	10 (5–0–5)	10 (5–0–5)
		**EAA^®^** (g/day) (g/intake)	---	---	25 (8.3–8.3–8.3)	25 (8.3–8.3–8.3)
		**Duocal^®^** (g/day) (g/intake)	---	30 (10–10–10)	30 (10–10–10)	---
		**Vitajoule^®^** (g/day) (g/intake)	---	---	---	60 (20–20–20)
Natural protein intake (g/day) *			25	27	35	45–50
Energy intake (Kcal/day) *^Δ^			1000	1300	1550	1950

* Natural protein and energy intake were estimated through Odimet^®^ (https://www.odimet.es/, Hospital Clínico Universitario de Santiago de Compostela, Santiago de Compostela, Spain; accessed on 1 December 2022), according to the patient’s 5-day food records. The composition of each of the products can be found in Supplementary Table S1. **^Δ^** Energy intake considers both natural sources and nutritional products. EAA: essential amino acid; MCT: medium chain triglyceride.

**Table 3:** Specific renal tests during the third trimester and after pregnancy.

	Reference Values	Gestational Week 33	Gestational Week 36	1–Year after Pregnancy
**Cr (mg/dL)**	**0.3–1.3**	0.44	0.43	0.44
**Cys C (mg/L)**	**0.6–1.1**	0.96	0.86	0.82
**eGFR (CKD–EPI [Cr])**	**≥90** **mL/min/1.73 m^2^**	136	137	135
**eGFR (CKD–EPI [CysC])**		87	101	107
**Cr clearance (mL/min/1.73 m^2^)**	**70–135**	126	116	106
**Ur protein (mg/24 h)**	**<150**	144	260↑	112
**Ur protein (mg/g Cr)**	**<200**	250↑	726↑	104

↑ Above the reference range. Cr: creatinine; Cys C: cystatin C; eGFR: estimated glomerular filtration rate; Ur: urinary.

**Table 4:** Hematological and hemostatic assessment before, during, and after pregnancy.

	Reference Values	Before Pregnancy	1st Trimester [1–13 Weeks]	2nd Trimester [14–27 Weeks]	3rd Trimester [28–37 Weeks]	1–Year after Pregnancy
**Blood cells**						
RBC count × 10^12^/L	**3.80–4.8**	4.69	4.43	2.55↓	3.52↓	4.94
MCV (fl)	**80–100**	90.4	87.9	90.2	91.0	91.4
MCH (pg)	**26.7–33.0**	27.9	28.1	29.4	29.7	28.4
Reticulocytes × 10^9^/L	**25.0–90.0**	66.5	–	36.9	75.7	81.0
Hemoglobin (mg/dL)	**12.0–15.0**	13.1	12.4	9.1↓	7.6↓	14.0
Hematocrit (L/L)	**0.36–0.46**	0.42	0.40	0.24↓	0.31↓	0.45
EPO (µmol/L)	**4.0–20.0**	–	–	–	629.0	15.8
WBC count × 10^9^/L	**4–11**	2.99↓	2.97↓	3.54↓	3.71↓	4.07
Platelets (EDTA) × 10^9^/L	**130–400**	104↓	142	108↓	83↓	117↓
Platelets (citrate) × 10^9^/L	**150–400**	–	–	Platelets aggregates	–	Platelets aggregates
**Micronutrients related to RBC function**						
Ferritin (ng/mL)	**15–200**	474↑	756↑	1807↑	1017↑	1256↑
RBC folate (ng/mL)	**250–1000**	265	246↓	264	177↓	447
Vit. B12 (pg/mL)	**250–1050**	714	773	1084	795	1002
Homocysteine (µmol/L)	**<15**	–	–	–	3.2	14.7
Copper (µg/dL)	**70–140**	39↓	61↓	131	138	66↓
Selenium (µg/dL)	**60–150**	43↓	47↓	58↓	50↓	62
Zinc (µg/dL)	**59–110**	140↑	225↑	84	55	107
**Hemolysis assessment**						
LDH (U/L)	**<234**	265↑	1740↑	1072↑	826↑	1144↑
Total bilirubin (mg/dL)	**<1.2**	0.7	0.9	0.5	0.3	0.5
Direct bilirubin (mg/dL)	**<0.6**	0.2	0.2	0.3	0.3	0.2
Haptoglobin (g/L)	**0.32–1**	<0.01	<0.01	<0.01	<0.01	<0.01
**Coagulation tests**						
Prothrombin time (s)	**9.9–13.6**	13.1	12.9	15.0	14.2	13.0
Prothrombin time (%)	**80–100**	86.2	86.3	68.1↓	68.4↓	92.7
aPPT (s)	**23.5–32.0**	24.6	24.4	26.3	29.7	25.6
INR		1.07	1.13	1.27	1.21	1.12

↓ Below and ↑ above the reference range. The median value for each trimester is represented: blood tests were performed every 4 months, except for the 2nd and 3rd trimesters (every 2 months). The 3rd trimester has an end at week 37.0 because labor was induced. aPPT: activated Partial Thromboplastin Time; EDTA: Ethylenediaminetetraacetic acid; EPO: erythropoietin; INR: International Normalized Ratio; LDH: lactate dehydrogenase; MCH: mean corpuscular hemoglobin; MCV: mean corpuscular volume; RBC: red blood cells; sec: seconds; Vit: vitamin; WBC: white blood cells.

## Data Availability

The data presented in this case report are available on request from the corresponding authors.

## Supplementary Materials

The following supporting information can be downloaded at: https://www.mdpi.com/article/10.3390/jcm12196405/s1, Table S1: Natural and dietary products provided throughout pregnancy.
