# Supplementary material for: Lysinuric Protein Intolerance and Its Nutritional and Multisystemic Challenges in Pregnancy: A Case Report and Literature Review
Source: J Clin Med. 2023 Oct 8;12(19):6405. doi: 10.3390/jcm12196405 (PMC10573933; doi:10.3390/jcm12196405)
Supplement: Supplementary file 1 [file jcm-12-06405-s001.zip › jcm-2637726-supplementary.pdf]

Journal of
Clinical Medicine

Supplementary Table S1. Natural and dietary products provided throughout pregnancy.

**Commercial multifruit juice concentrate: Pineapple, Orange, Apple, Apricot, Grapes (Eckes-Granini Iberica, S.A.U, Barcelona, Spain)**
Nutritional information is expressed per 100mL.
Energy 52.0Kcal; Carbohydrates 11.9g; Proteins 0.5g; Fat 0.1g.

**L-citrulline (Nutricia N.V., Zoetermeer, The Netherlands)**
A powdered form of the amino acid L-citrulline.
Nutritional information is expressed per 100g.
Energy 359.0kcal; Carbohydrates 0.0g; Protein equivalents 89.7g; L-citrulline 100.0 g; Fat 0.0g.

**Medium-chain triglycerides oil (Nutricia N.V., Zoetermeer, The Netherlands)**
A liquid containing a mixture of medium-chain triglycerides (MCT).
Nutritional information is expressed per 100mL.
Energy 855.0Kcal; Carbohydrates 0.0g; Protein equivalents 0.0g; Fat 95.0g.

**Duocal® (Nutricia N.V., Zoetermeer, The Netherlands)**
A high-energy powdered product that contains a dual energy source of carbohydrates and fat.
Nutritional information is expressed per 100g.
Energy 492.0Kcal; Carbohydrates 72.7g; Fat 22.3 g (saturated fat 10.4 g).

**Vitajoule® (Vitaflo a Nestlé Health Science Company, Vevey, Switzerland)**
A carbohydrate powder supplement with no fat content.
Nutritional information is expressed per 100g.
Energy 380.0 Kcal; Carbohydrates 95.0 g; Fat 0.0g.

**EAA Supplement® (Vitaflo a Nestlé Health Science Company, Vevey, Switzerland)**
Unflavored powder mix of essential amino acids and carbohydrates.<br>*Nutritional information is expressed per 100g.*<br>Energy 265.0 Kcal; Protein equivalents 40.0 g; Carbohydrates 26.0g; Fat 0.1g.<br>L-cysteine 1.9g; L-phenylalanine 3.6g; L-histidine 1.9g; L-leucine 10.5g; L-lysine 7.1g; L-isoleucine 6.1g; L-methionine 1.9g; L-tyrosine 5.0g; L-threonine 4.4g; L-tryptophan 1.4g; L-valine 7.7 g.
